# Understanding Enhanced Ionic Conductivity in Composite Solid‐State Electrolyte in a Wide Frequency Range of 10^–2^–10^10^ Hz

**DOI:** 10.1002/advs.202200213

**Published:** 2022-04-23

**Authors:** Kai‐Lun Zhang, Na Li, Xu Li, Jun Huang, Haosen Chen, Shuqiang Jiao, Wei‐Li Song

**Affiliations:** ^1^ Institute of Advanced Structure Technology Beijing Institute of Technology Beijing 100081 P. R. China; ^2^ Institute of Theoretical Chemistry Ulm University Ulm 89069 Germany; ^3^ State Key Laboratory of Advanced Metallurgy University of Science and Technology Beijing Beijing 100083 P. R. China

**Keywords:** electrical response, ionic conductivity, multiscale mechanism, solid state electrolytes

## Abstract

The ionic conductivity of composite solid‐state electrolytes (SSEs) can be tuned by introducing inorganic fillers, of which the mechanism remains elusive. Herein, ion conductivity of composite SSEs is characterized in an unprecedentedly wide frequency range of 10^–2^–10^10^ Hz by combining chronoamperometry, electrochemical impedance spectrum, and dielectric spectrum. Using this method, it is unraveled that how the volume fraction *v* and surface fluorine content *x*_F_ of TiO_2_ fillers tune the ionic conductivity of composite SSEs. It is identified that activation energy *E*_a_ is more important than carrier concentration *c* in this game. Specifically, *c* increases with *v* while *E*_a_ has the minimum value at *v *= 10% and increases at larger *v*. Moreover, *E*_a_ is further correlated with the dielectric constant of the SSE via the Marcus theory. A conductivity of 3.1×10^–5^ S cm^−1^ is obtained at 30 °C by tuning *v* and *x*_F_, which is 15 times higher than that of the original SSE. The present method can be used to understand ion conduction in various SSEs for solid‐state batteries.

## Graphical abstract

A broadband method that combines chronoamperometry, electrochemical impedance spectrum, and dielectric spectrum is developed to characterize ion conductivity of composite solid‐state electrolytes (SSEs) in a wide range of 10^–2^–10^10^ Hz. According to the competition between interface and bulk conduction, optimal volume fraction of inorganic fillers is calculated to achieve a maximum ionic conductivity of composite SSEs.

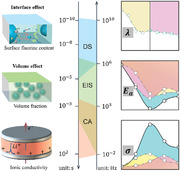

## 1 Introduction

Solid‐state lithium‐ion batteries are one of promising candidates for next‐generation energy storage devices due to high energy density and safety.^[^ ^1^, ^2^, ^3^, ^4^, ^5^ ^]^ Fast ion transport in solid‐state electrolytes (SSEs) is essential for practical solid‐state lithium‐ion batteries (LIBs).^[^ ^6^, ^7^, ^8^, ^9^, ^10^ ^]^ To achieve high ionic conductivity in the SSEs, current approaches include addition of soluble lithium salts,^[^ ^11^, ^12^ ^]^ oligomer plasticizers,^[^ ^13^, ^14^, ^15^ ^]^ and inorganic fillers.^[^ ^16^, ^17^ ^]^ Specifically, ion conductivity of composite SSEs can be improved by using a suitable lithium salt with both high oxidation stability and high dissociation degree.^[^ ^12^ ^]^ In addition, oligomer polymers, including poly(ethylene glycol) (PEO)‐based polymers,^[^ ^15^ ^]^ ethylene carbonates,^[^ ^18^ ^]^ propylene carbonates^[^ ^14^ ^]^ among others, have been added in the matrix of the composite SSEs as the plasticizer. The added plasticizer can decrease the crystallinity of PEO and the activation barrier of ion migration, thus increasing ion mobility.^[^ ^13^ ^]^ Besides, adding inorganic fillers is another method to improve ionic conductivity of SSEs.^[^ ^19^, ^20^, ^21^, ^22^, ^23^ ^]^ Owing to the fast interfacial ion transport,^[^ ^24^ ^]^ both ionically conductive ceramics^[^ ^25^ ^]^ and electrically insulating oxides^[^ ^26^, ^27^, ^28^ ^]^ have been considered as the inorganic fillers for fast ion transport in the composite SSEs. Interestingly, the volume of filler has a significant effect on the ionic conductivity.^[^ ^29^, ^30^, ^31^, ^32^, ^33^, ^34^ ^]^ Therefore, the effects of volume/mass fraction and microscopic structure of the fillers have been systemically studied.^[^ ^35^, ^36^, ^37^, ^38^ ^]^ However, the structure–property relationship of ion transport in the composite SSEs is still unclear.

Characterization of ion migration in the SSEs is a challenging task that needs to cover a broad frequency range spanning from 10^–2^ to 10^10^ Hz. In the frequency range between 10^6^ and 10^10^ Hz, ion conduction under an external alternating electric field can be characterized using dielectric spectrum (DS).^[^ ^35^ ^]^ In the frequency range between 10^4^ and 10^9^ Hz, solid‐state nuclear magnetic resonance spectrum can be used to separate the states and dynamics of ion conduction in interfacial and bulk phases.^[^ ^39^, ^40^, ^41^ ^]^ Anisotropic interactions between conducting ions and the interface can be modulated by the external magnetic field. For the bulk conductivity of SSEs, electrochemical impedance spectrum (EIS, 10^–2^–10^6^ Hz) coupled with blocking electrodes is the most widely used method.^[^ ^42^ ^]^ In the frequency range between 10^–2^ and 10^3^ Hz the ionic hopping occurs in the bulk and diffusion of Li^+^ at the grain boundary. Additionally, the low frequency bulk conductivity can be measured though chronoamperometry (CA),^[^ ^43^ ^]^ which is a time domain method and has a typical frequency range of 10^–2^–10^3^ Hz when transformed to the frequency domain.

**Figure 1:**
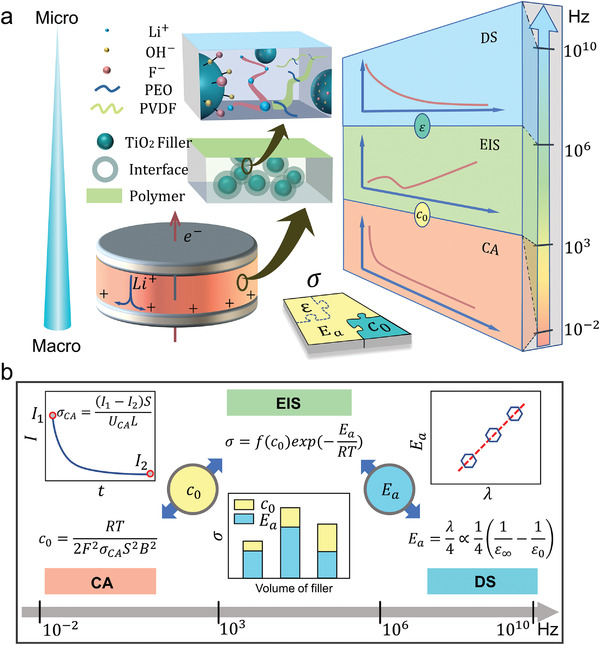
Conceptual illustration of the proposed multispectral analytical method for solid‐state ionic transport: (a) schematic diagram of the proposed method that combines CA, EIS, and DS spectra; b) Relationships between parameters obtained from CA, EIS, and DS (*σ*_CA_: ionic conductivity from CA, *c*: carrier concentration, *σ*: ionic conductivity from EIS, *E*_a_: activation energy, *ε*_0_ and *ε*_∞_: static and optical dielectric constants from DS); Key parameters including *c*_0_ and *E*_a_ are transferred between these three spectra; In the used relationship, *B* is the Warburg constant, *λ* is the solvent reorganization factor, *S* is the area of ion‐blocking electrode, *U*_CA_ is the applied DC voltage of CA, and *L* is the thickness of composite SSEs.

Though each method could provide crucial insights, obtaining a more comprehensive picture of ion conduction in composite SSEs requires the combination of multiple methods. This is the main motivation of this work. DS,^[^ ^35^ ^]^ EIS,^[^ ^42^ ^]^ and CA^[^ ^43^ ^]^ are combined to characterize ion conduction in several types of SSEs, with the matrix made of PEO and poly(vinylidene fluoride)(PVDF), fillers made of pristine TiO_2_ or surface fluorinated TiO_2_, and lithium salt made of lithium‐bistrifluoromethanesulfonimidate (LiTFSI) (**Figure** **1**a). Several key quantities related to ion conduction, including the ionic conductivity, activation energy, carrier concentration and dielectric constant, are obtained, and their dependence on the volume and microscopic surface structure of fillers is then investigated (Figure 1b). The data are then discussed in a view of interactions between bulk and interfacial pathways of ion conduction. The fundamental understanding is then exploited to improve the ionic conductivity of composite SSEs by combining changing volume fraction and surface fluorine content. The approach developed here is instrumental to design and optimization of highly conductive SSEs for solid‐state LIBs.

## 2 Results and Discussion

**Figure 2:**
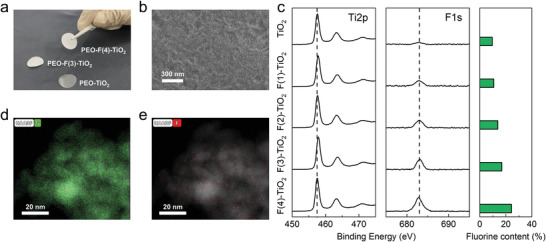
Fluorination of the added TiO_2_ filler: a) Photographic diagram and b) cross‐sectional scanning electron microscopy (SEM) image of composite SSEs; c) X‐ray photoelectron spectrum (XPS) results and surface fluorine content of TiO_2_ fillers; d) the high‐angle annular dark‐field (HAADF) mapping for e) Ti and f) F elements in the surface of TiO_2_ filler.

The proposed method combines CA, EIS, and DS in a total frequency range of 10^–2^–10^10^ Hz. Ion conduction in composite SSEs has been characterized through the proposed method. The composite SSEs are fabricated by adding TiO_2_ fillers into polymer matrix made of PEO and PVDF (**Figure** **2**a). A constant amount lithium salt, i.e., LiTFSI, with a ratio of Li^+^ : EO = 1:15 was added in different SSEs. Ionic conductivity of composite SSEs with different amount of added TiO_2_ fillers was measured. The effect of filler volume is examined at 5%, 10%, 15%, and 20% volume fractions *v* of TiO_2_ fillers. Given a volume fraction *v* of fillers, the surface fluorine content *x*_F_ is tuned through fluorination using hydrofluoric acid solution (**Table** **1**), which has been verified through X‐ray photoelectron spectroscopy (XPS) and transmission electron microscopy (TEM) results (Figure 2).

**Table 1:** Peak index ratio of Ti2p: F1s from XPS results of fluorinated TiO_2_ nanofillers

Sample	Ingredient amount		Rate of peak index (Ti 2p: F1s)
	TiO_2_ [g]	HF [wt%]	
TiO_2_	1.59	0.0	9.45:1
F(1)‐TiO_2_	1.59	8.7	8.31:1
F(2)‐TiO_2_	1.59	16.0	6.25:1
F(3)‐TiO_2_	1.59	22.0	4.92:1
F(4)‐TiO_2_	1.59	27.0	3.12:1

**Figure 3:**
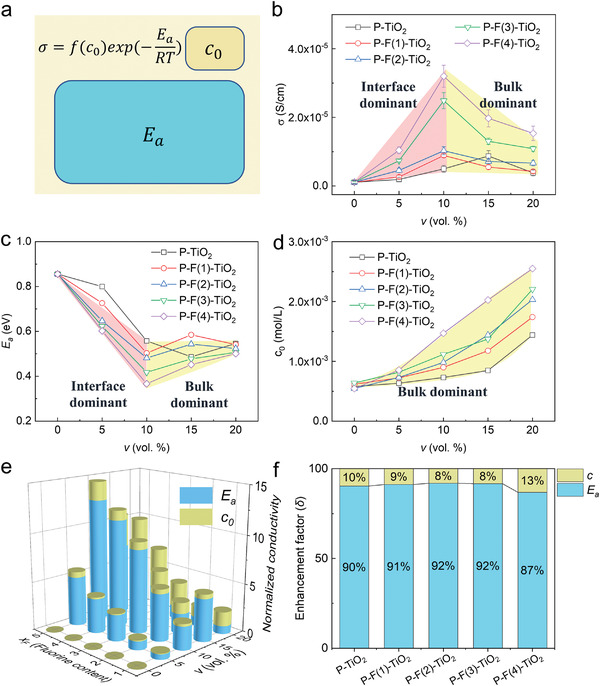
Effect of filler volume *v* on ionic conductivity of composite SSEs: a) Relationship between carrier concentration *c*, activation energy *E*_a_ and ionic conductivity *σ*; b) *σ*, c) *E*_a_, and d) *c* of SSEs with different volume fractions *v* and surface fluorine contents *x*_F_ of TiO_2_ fillers at 30 °C. e) Normalized conductivity $\frac{\sigma_{i}}{\sigma_{0}}$ of composite SSEs in comparison with neat polymer SSEs. (f) Normalized enhancement factor of *c* and *E*_a_ to the ionic conductivity.

The ionic conductivity of composite SSEs can be expressed as^[^ ^44^ ^]^

(1) $$\sigma \;=\frac{c_{0}F\tau^{2}}{RT}\exp\left(-\frac{E_{a}}{RT}\right)$$

where *c* is carrier concentration, *E*_a_ is the activation energy, *τ* is the mean free path. *R*, *T*, *F*, and *q* are gas constant, temperature, Faraday constant, and the charge of elementary charge, respectively. Ionic conductivity, activation energy, and carrier concentration of composite SSEs are obtained though combined analysis of CA and EIS (**Figure** **3**). The ionic conductivity first increases and then decreases with increased *v* (Figure 3b). The maximum conductivity of P‐TiO_2_ composite SSEs, 8.7 × 10^–6^ S cm^−1^, is obtained at *v* = 15%. Polymer/filler interfaces were introduced into the electrolyte after adding inorganic TiO_2_ fillers. These interfaces possess higher ionic conductivity than the bulk polymer SSEs, resulting in increased conductivity of composite SSEs.^[^ ^24^ ^]^ With an increased *x*_F_ of TiO_2_ fillers, the optimal volume fraction *v*_optimal_ of fluorinated TiO_2_ filler decreases to 10%. The maximum ionic conductivity of P‐F(4)‐TiO_2_ increases to 3.1 × 10^–5^ S cm^−1^ at 30 °C and the minimum activation energy is 0.34 eV (Figure 3c). A higher ionic conductivity can be achieved when ionic conductive filler like Li_6.4_La_3_Zr_1.4_Ta_0.6_O_12_ was added into PEO electrolyte.^[^ ^16^ ^]^ *E*_a_ is calculated using the Arrhenius equation (Figure S3, Supporting Information). The studied temperature range is 20–60 °C, which is lower than the glass‐transition temperature *T*_g_ of PEO (65–70 °C). The crystalline change of PEO can be ignored and the slope of log(*σ*) ≈ 1000/*T* curve keeps constant in Figure S3 in the Supporting Information. Thus, Arrhenius equation is more suitable than Vogele–Tammanne–Fulcher equation to calculate activation energy *E*_a_. The carrier concentration of composite SSEs can be calculated

(2) $$c\;=\frac{RT}{2F^{2}\sigma_{\mathrm{CA}}S^{2}B^{2}}\;$$

where *σ*_CA_ is the diffusion‐dominated ionic conductivity (Experiment Section),^[^ ^43^ ^]^ *B* is the Warburg constant derive from EIS results (Figure S2, Supporting Information). Equation (2) is derived from Nernst–Einstein equation (derivation details is given in the Supporting Information). A maximum concentration of 0.0026 mol L^−1^ is achieved in P‐F(4)‐TiO_2_ with 20 vol% TiO_2_ (Figure 3d). The carrier concentration *c* increases with the added amount of TiO_2_ filler because the introduced polymer/filler interfaces have higher *c* than bulk polymer due to electric double layer effects.^[^ ^45^, ^46^ ^]^ Increased carrier concentration and higher activation energy constitute two critical factors when tuning the ionic conductivity through changing the amount of TiO_2_ fillers.

The contributions of carrier concentration *c* and activation energy *E*_a_ to ionic conductivity are quantified in Figure 3e. The normalized enhancement factor of *c* was calculated as

(3) $$\delta_{c}=\frac{c_{i}}{c_{0}}\;\frac{\sigma_{0}}{\sigma_{i}}$$

where *c*_0_ and *σ*_0_ are carrier concentration and ionic conductivity of bulk polymer SSEs, *c*_i_ and *σ*_i_ are carrier concentration and ionic conductivity of composite SSEs. The normalized enhancement factor of *E*_a_ is $\delta_{E_{a}}=\;1-\delta_{c}$. $\delta_{E_{a}}$ and *δ*_c_ are used to compare the influence of *c* and *E*_a_ on the tuned conductivity (Figure 3f). At the optimal volume fraction (*v*_optimal_ = 10%) of filler, $\delta_{E_{a}}$ is larger than 85%. The reduced *E*_a_ plays a more critical role than increased *c* in the enhanced ion conduction of composite SSEs.

**Figure 4:**
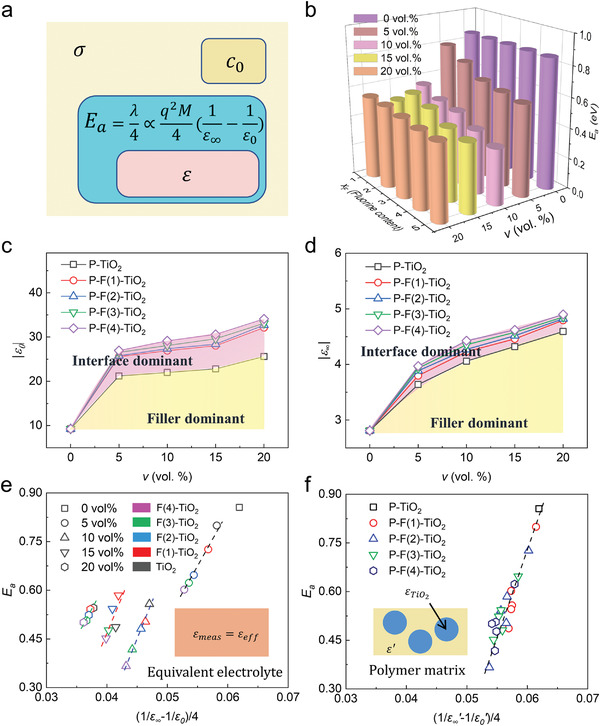
Interfacial effect of filler on activation energy in the composite SSEs: a) Relationship between activation energy, and dielectric constant; b) activation energy of SSEs with different TiO_2_ fillers; c) optical dielectric constant and d) static constant of composite SSEs; e) relationship between activation energy *E*_a_ and dielectric constant factor $\frac{1}{4}\left(\frac{1}{\varepsilon_{\infty }}-\frac{1}{\varepsilon_{0}}\right)$ calculated from dielectric constant of SSEs; f) dielectric constant factor of polymer matrix $\frac{1}{4}\left(\frac{1}{{\varepsilon_{\infty }}^{\prime }}-\frac{1}{{\varepsilon_{0}}^{\prime }}\right)$ versus *E*_a_.

The effect of changed *x*_F_ of filler on the activation energy of ion conduction is illustrated by the combination of EIS and DS spectra (**Figure** **4**). Optical and static dielectric constant (Figure 4c,d) are obtained through dielectric spectrum of composite SSEs. Due to the limitation of measurement accuracy, the highest (10^6^ Hz) and lowest (10^10^ Hz) frequency dielectric constant are selected as optical and static dielectric constant, respectively. In the yellow part of dielectric constant plots, both optical and static dielectric constant of P‐TiO_2_ increased with increasing *v*. The increased dielectric constant is attribute to higher dielectric constant of TiO_2_ (≈30 at 10^6^ Hz, Figure S4f, Supporting Information) than polymer matrix (≈10 at 10^6^ Hz, Figure S4a, Supporting Information). In the pink part of dielectric constant plots, a further increased dielectric constant of composite SSEs results from increased *x*_F_ of TiO_2_ filler.

We try to understand the relationship between increased dielectric constant of composite SSEs and decreased activation energy of ion conduction (Figure 4b) in a view of interfacial electron transfer. In Marcus theory, energy barrier of charge transfer activation energy is decided by the solvent reorganization energy, which is closely related to variation of dielectric constant of electrolytes:^[^ ^47^ ^]^

(4) $$E_{a}=\frac{\lambda }{4}\;\propto \frac{q^{2}M}{4}\left(\frac{1}{\varepsilon_{\infty }}-\frac{1}{\varepsilon_{0}}\right)$$

where *λ* represents the solvent reorganization factor, *M* is the distance of charge transfer. *ε_∞_* and *ε*_0_ are optical and static dielectric constant of SSE, respectively. According to Equation (4), linear relationships between activation energy *E*_a_ and dielectric constant factor $\frac{1}{4}\left(\frac{1}{\varepsilon_{\infty }}-\frac{1}{\varepsilon_{0}}\right)$ can be verified at fixed *v* (Figure 4e). The decreased dielectric constant factor results from increased *x*_F_ of TiO_2_ fillers, leading to reduced activation energy of ion conduction. With increased *v*, an obviously decreased dielectric constant factor from 0.062 to 0.036 is observed. This phenomenon is contributed by the high dielectric constant of primary TiO_2_ fillers (Figure S4f, Supporting Information). The contribution of primary TiO_2_ fillers should be excluded because that the conduction ions cannot path through the bulk of TiO_2_ particles. Effective medium theory (inset of Figure 4e,f) was used to calculate dielectric constant of polymer matrix *ε^′^* from the measured dielectric constant of composite SSEs *ε*:^[^ ^48^ ^]^

(5) $$\varepsilon \;=\varepsilon^{\prime }\;v+\varepsilon_{\mathrm{Ti}O_{2}}\left(1-v\right)$$

where *ε^’^* represents dielectric constant of polymer matrix, $\varepsilon_{\mathrm{Ti}O_{2}}$ is the dielectric constant of primary TiO_2_ fillers. After replacing *ε^’^* with *ε*, the influence of changed *v* is excluded. A quasi‐linear relationship between activation energy and dielectric constant factor of polymer matrix is extracted (Figure 4f). The slope of normalized relationship presents similar transferred charge quantity and distance of ion conduction. Thus, Marcus theory of interfacial electron transfer is applied to describe ion conduction. It is helpful to reveal the influence of increased *x*_F_ of TiO_2_ filler on the reduced *E*_a_ of interfacial conduction.

**Figure 5:**
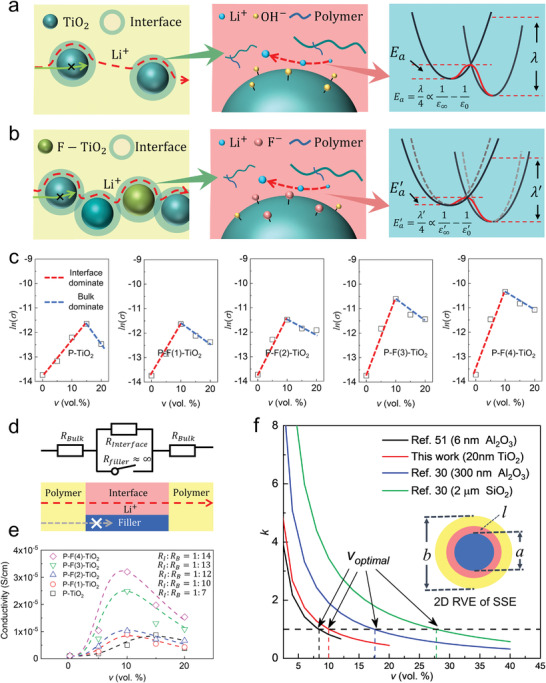
The mechanisms of filler volume and interfacial effects for achieving high ionic conductivity of SSEs: the effect of filler volume refers to manipulating the filler loading in the electrolyte matrices, and the interfacial effect refers to utilizing functionalization to modify the surface fluorine content *x*_F_ of fillers prior to adding into the electrolyte matrices. In a) P‐TiO_2_ and b) P‐F‐TiO_2_, mechanism scheme of the filler volume effect on ion conduction (left), the interfacial effect on ion conduction (middle), and decreased *E*_a_ in the interfacial effect of filler (right); c) the conductance volcano plots of *v* dependent ionic conductivity of composite SSEs; d) series equivalent circuit model (upper) and effective conducting model of ions (bottom); e) fitted relationships between *σ*_total_ and *v* according to Equation (6); f) literature comparison of *k* and representative element volume (RVE) of composite SSEs (inset), the composite SSEs has a maximum conductivity when *k* approaches 1.

The mechanism of filler volume and interfacial effects is demonstrated in **Figure** **5**. Effect of filler volume refers to manipulating the filler loading (e.g., pristine TiO_2_ in this study) in the electrolyte matrices, and interfacial effect refers to utilizing functionalization to modify the surface of fillers (e.g., different fluorinated TiO_2_ in this study) prior to adding into the electrolyte matrices. Ion conduction in the polymer/filler interface is faster than that in the bulk electrolyte due to the nature of electric double layer.^[^ ^24^ ^]^ After adding more inorganic fillers, highly conductive channels could be constructed by the polymer/ filler interface (left of Figure 5a,b). The effect of filler volume presents an increased total ionic conductivity of composite SSEs with inorganic fillers.

The mechanism of enhanced interfacial conduction is exhibited in the middle of Figure 5a,b. Reduced *E*_a_ of interfacial ion conduction is mainly affected by increased *x*_F_ of TiO_2_ fillers. The surface ions of the pristine TiO_2_ particles mainly refer to the functional groups of OH^–^,^[^ ^49^ ^]^ Conductive ions would be influenced by these OH^–^ and the side chain of polymer in the interface. After fluorination of TiO_2_ fillers, F^–^ would partially replace the functional groups on the surface of TiO_2_. Because of the enhanced surface polarity of F^–^, increase dielectric constant of composite SSEs was measured. Decreased solvent reorganization factor *λ* is obtained according to the calculated dielectric constant factor from measured dielectric constants (Figure 4e). The curvature of the potential energy surface of charge transfer in P‐F‐TiO_2_ is smaller than P‐TiO_2_ (right of Figure 5a,b), resulting in lower energy barrier of interfacial conduction. Thus, ion conductivity of interface region *σ*_I_ increases with increased *x*_F_ of TiO_2_ fillers (Figure 5e).

A simple series equivalent circuit model has been established to describe *v* dependent ion conductance (Figure 5d). The total conductivity of the composite SSEs can be written as

(6) $$\sigma_{\mathrm{total}}=\sigma_{0}\;+w\frac{1}{{\left(\sigma_{I}\eta \right)}^{\alpha }+{\left(\sigma_{B}\left(1-\eta \right)\right)}^{\beta }}$$

where *η* is the volume fraction of interface region, *w* represents the normalized factor of conductivity (ranges from 2.5×10^–5^ to 7.5×10^–5^ S cm^−1^), *σ*_0_ is the ionic conductivity of bulk polymer electrolyte, *σ*_I_ and *σ*_B_ are the ionic conductivity of interface and bulk phases of polymer matrix. *α* and *β* are the Bruggeman correlation of conductivity in porous media.^[^ ^50^ ^]^ The relationship between *σ*_total_ and *v* has been fitted using Equation (6) (Figure 5e). Specific values of parameters in the fitted equation are provided in Table S1 in the Supporting Information). A initial conductivity ratio of *σ*_B_ : *σ*_I_ = 1:7 is used to fit the *v* dependent ionic conductivity of P‐TiO_2_. *σ*_I_ decreases due to increased *x*_F_ of TiO_2_ fillers. The used conductivity ratios of *σ*_B_ : *σ*_I_ increased to 1:10, 1:12, 1:13, and 1:14 for P‐F(1)‐TiO_2_, P‐F(2)‐TiO_2_, P‐F(3)‐TiO_2_, and P‐F(4)‐TiO_2_, respectively. *v*_optimal_ experiences an undetectable decrease when increasing the interfacial conductivity *σ*_I_. This may explain the difference of *v*_optimal_ between the prime TiO_2_ filler and fluorinated F‐TiO_2_ fillers. Unfortunately, the changed *v*_optimal_ of fluorinated fillers has not been observed due to a low regulating accuracy of *v*.

The competition in between bulk *σ*_B_ and interfacial conduction *σ*_I_ is discussed though conductance volcano plots of composite SSEs (Figure 5c). With fixed *x*_F_ of filler, there is a *v*_optimal_ of added filler to obtain the maximum conductivity of composite SSEs. The increased conductivity is dominated by increased fraction of interfacial conduction with low *v*. When *v* is larger than *v*_optimal_, overlap of interface regions should not be ignored. The fraction of *σ*_B_ increases and *σ*_I_ decreases with increased *v*, resulting in decreased total conductivity of the composite SSEs. According to the competition between bulk and interfacial conduction, a dimensionless variable *k* is used to calculate *v*_optimal_ of inorganic filler:

(7) $$k\;=\frac{\pi \left(b^{2}-a^{2}\right)}{\pi {\left(a+l\right)}^{2}-\pi a^{2}}\;=\frac{\left(1-v\right)b^{2}}{{\left(a+l\right)}^{2}-vb^{2}}\;$$

where *a*, *b*, and *l* are radius of inorganic filler, radius of representative element volume (RVE) of composite SSEs, and thickness of filler/polymer interface, respectively (inset of Figure 5f). The specific area of TiO_2_ fillers can be tuned through increased radius *a*, which would change the ratio of TiO_2_/PEO interface and increase the optimized volume ratio *v* of TiO_2_ fillers. The volume fraction of filler is *v = *(*a/b*)^2^. Thickness of interface layer is calculated through Debye length $l\;=\sqrt{\frac{\varepsilon k_{B}T}{c_{0}q^{2}}}\;$. *k*_B_ is the Boltzmann constant. The dimensionless variable *k* can be simplified as

(8) $$k\;=\frac{1-v}{\left(\gamma^{2}+2\gamma \right)v}\;$$

where *γ* is the ratio of interface thickness and filler radius and *γ *= *l*/*a*. When *k* = 1, corresponding *v* equals to *v*_optimal_. The composite SSEs possesses a maximum *σ*_total_. The interfacial phase occupies a maximum volume fraction of polymer matrix without overlapped region:^[^ ^44^ ^]^

(9) $${\left(1+\gamma \right)}^{2}\;v_{\mathrm{optimal}}=\;1$$

*v*_optimal_ is closely related to the interface thickness and filler radius. In our work, the thickness of interface layer is ≈ 43 nm in the fabricated composite SSEs, when *c* = 2 × 10^2^ mol L^−1^ and *ε* = 26. The calculated *v*_optimal_ ≈ 10%, which is consistent with the experiment results (Figure 5e). Moreover, the *v*_optimal_ of TiO_2_ and fluorine TiO_2_ fillers has the same physical meaning with the critical value of inorganic fillers in other literatures (Figure 5f).^[^ ^30^, ^51^ ^]^ The composite SSEs have a maximum conductivity when the polymer matrix is totally occupied by the polymer/filler interface. This physical scenario of *v*_optimal_ of added inorganic fillers is critical to promote the ionic conductivity of composite SSEs though competition between bulk and interfacial conduction.^[^ ^44^ ^]^

The as‐established analytic method in this study highlights a platform for understanding the solid‐state ion conduction in a wide frequency range of 10^–2^–10^10^ Hz. The interconnections among physical parameters including *σ*, *c*, *E*_a_, and dielectric constant have been established through the joint analysis of CA, EIS, and DS. Such method could be used to obtained the interaction between separated spectra, by which the interconnections of ion conduction at different time‐scales could be well understood.^[^ ^52^ ^]^ The interconnections between key parameter analysis from different scales apparently provide new physical implication, offering a unique aspect based on the joint analytical method. In addition, the multispectral analytical method can be extended to characterization of other types of electrically insulating dielectrics, along with analyzing the failure mechanism of composites with applied voltage. Combining with percolation model for electrical conduction,^[^ ^53^ ^]^ a general measuring method for multiscale charge transfer model including ions and electrons is highly expected.

## 3 Conclusion

In summary, we developed a combined analysis method for studying solid‐state ion conduction in composite SSEs in a wide frequency range of 10^–2^–10^10^ Hz. Critical parameters of ion conduction have been determined from the joint analysis of CA, EIS, and DS spectra. At 30 °C, the maximum total ionic conductivity of 3.1×10^–5^ S cm^−1^ was obtained a volume fraction of added TiO_2_ of 10%, which is over 15 times larger than the conductivity of polymer SSEs without inorganic fillers. The contributions of decreased activation energy and increased carrier concentration to the enhanced ionic conductivity were separated. Specifically, the contribution of decreased activation energy is much larger than that of increased carrier concentration. A linear relationship between the activation energy and the dielectric constant was established based on Marcus theory. The dielectric constant of composite SSEs is decreased with increased surface fluorine content of TiO_2_ filler, resulting in decreased activation energy and enhanced interfacial conduction. Moreover, ion conduction in composite SSEs is explained using an equivalent circuit model based on volume and interfacial effects of fillers. The competition between bulk and interfacial conduction leads to an optimal volume fraction of added inorganic fillers. A physical dimensionless variable *k* is proposed to calculate the optimal volume fraction. These results provide a new platform for understanding the mechanism of ion conduction in the composite SSE for solid‐state LIBs.

## 4 Experimental Section

### Sample Preparation—Fluorination of TiO_2_ Nanofillers

The amorphous TiO_2_ nanoparticles are bought from Nanjing XFNANO Materials Tech Co., Ltd. D50 of the TiO_2_ nanoparticle is 20 nm. After washed with deionized water and ethanol, the dried TiO_2_ particles are soaked in hydrofluoric acid solution for 10 h at room temperature. The concentration of hydrofluoric acid solution was tuned for the manipulation of surface fluorine content of TiO_2_. Four level of hydrofluoric acid concentration including 8.7, 16, 22, and 27 wt% were chosen. The surface fluorine content of TiO_2_ particles was characterized though XPS (Figure 2c). Corresponding fluorinated TiO_2_ nanoparticles are named F(1)‐TiO_2_, F(2)‐TiO_2_, F(3)‐TiO_2_, and F(4)‐TiO_2_ according to the peak ratio of Ti2p: F1s (Table 1).

### Sample Preparation—Fabrication of Composite SSE

Poly(ethylene oxide) (PEO, molecular weight (MW) 6 000 000) and polyvinylidene fluoride (PVDF, MW 455 000) were obtained from ShangHai EKEAR Bio@Tech Co. Ltd. and Sigma‐Aldrich Co. Ltd. 0.12 g PEO, 0.1 g PVDF, and preconcerted amount of TiO_2_ nanofiller were dissolved in 10 mL acetonitrile. The volume ratio of TiO_2_ was calculated after considering the density of PEO (0.93 g cm^−3^), PVDF (1.78 g cm^−3^), and TiO_2_ (4.26 g cm^−3^). After a 10 h stirring at 60 ℃, desire amount of lithium lithium‐bis(trifluoromethosulfonimide) (ether oxygen: lithium ion = 15:1) was added into the fully crosslinked mixture. A continued stirring was carried out until the lithium salt completely dissolved. The mixture was dried on a cleared glass plate for a week at 60 ℃. Finally, PEO‐TiO_2_ SSEs with a thickness of ≈ 30 µm were fabricated after being cut into circle. All these production procedures were carried out in a glovebox.

### Materials Characterization

XPS characterized on an ULVAC‐PHI instrument. Scanning electron microscopy (SEM) images were acquired on a Hitachi S4800 FE‐SEM system. TEM analyses were performed on a Hitachi HD‐2000 Scanning‐TEM system and a Hitachi H‐9500 TEM system, coupled with the use of carbon‐coated copper grids. TiO_2_ powder samples were dissolved with ethyl alcohol, followed by dropped on the copper grids. TEM specimens were obtained after dried at room temperature.

### Multispectrum Measurement—CA

A Bio‐logic AC electrochemical workstation is used for chronoamperometry measurement. A constant voltage with an amplitude of 100 mV is applied for 150 s in the measurement. The fabricated SSEs were clamped with two blocking stainless steel electrodes. The area of circular Ag coated stainless steel electrodes was 2 cm^2^. The original voltage‐current curve was recorded for the calculation of conductivity *σ*_CA_. The ionic conductivity *σ*_CA_ was calculated form the CA spectra (Figure S1, Supporting Information) of composite SSEs according to^[^ ^43^ ^]^

(10) $$\sigma_{\mathrm{CA}}=\frac{\left(I_{1}-I_{2}\right)S}{U_{\mathrm{CA}}L}\;$$

where *S* is the area of ion blocking electrodes, *U*_CA_ is the applied DC voltage, and *L* is the thickness of composite SSEs. The unsteady current and signal to noise ratio of Chronoamperometry have been listed in Table S2 in the Supporting Information.

### Multispectrum Measurement—EIS

The impedance spectrum was obtained though the Bio‐logic AC electrochemical workstation. The measurement frequency ranges from 10^–2^ Hz to 10^6^ Hz. The amplitude of alternating signal is 10 mV. The samples were connected with Ag coated blocking electrodes, putting into a thermostat for EIS measurement at different temperature. Warburg constant was obtained from the low frequency range results in EIS spectrums. After plot the real impedance versus angular frequency (*ω* ^–1/2^), the Warburg constant *B* was fitted by the slope of curve.

### Multispectrum Measurement—DS

The dielectric spectrums were measured using a Keysight E5071C vector network analyzer. Electromagnetic wave with an amplitude of 10 mV was exited for the dielectric spectrum measurement. The working frequency ranges from 10^6^ to 10^10^ Hz. The nonsolidified PEO‐TiO_2_ mixture was pouring into metal mold, getting circular composite SSE after dried for 24 h at room temperature. The inside and outside radiuses are 3 and 7 mm of circular SSE, respectively. The dielectric constant represents the magnitude of complex permittivity measured from DS method.

### Multispectrum Measurement—Parameters Calculation

EIS data were fitted with an electric circuit model given in Figures S2 and S3a in the Supporting Information, resulting in ionic conductivity and Warburg constant. By analyzing ionic conductivity at a series of temperatures using the Arrhenius equation, activation energy of different SSEs (Figure S3, Supporting Information) is obtained. When using a series equivalent circuit of *R*_e_ and *R*_i_, a high electric conductivity (≈10^–2^ S cm^−1^) of PEO electrolyte is calculated. When using a series equivalent circuit of *R*_e_ and *R*_i_, a low electric conductivity (≈10^–10^ S cm^−1^) of PEO electrolyte is calculated. According to the result of CA (Figure S1, Supporting Information), the electric conductivity of PEO electrolyte is low than 10^–8^ S cm^−1^. Thus a parallel equivalent circuit of *R*_e_ and *R*_i_ is used in the equivalent circuit for EIS (Figure S3a, Supporting Information). In addition, dielectric constant *ε* is acquired via the measured complex permittivity in DS spectrum (Figure S4, Supporting Information). The highest (10^6^ Hz) and lowest (10^10^ Hz) frequency dielectric constant were selected as optical and static dielectric constant, respectively.

## Conflict of Interest

The authors declare no conflict of interest.

## Supporting information

Supporting Information

## Data Availability

The data that support the findings of this study are available in the supplementary material of this article.
