# Supplementary material for: Understanding Enhanced Ionic Conductivity in Composite Solid‐State Electrolyte in a Wide Frequency Range of 10–2–1010 Hz
Source: Adv Sci (Weinh). 2022 Apr 23;9(18):2200213. doi: 10.1002/advs.202200213 (PMC9218661; doi:10.1002/advs.202200213)
Supplement: Supplementary file 1 — Supporting Information [file ADVS-9-2200213-s001.pdf]

ADVANCED SCIENCE
Open Access

Supporting Information

for *Adv. Sci.*, DOI 10.1002/advs.202200213

Understanding Enhanced Ionic Conductivity in Composite Solid-State Electrolyte in a Wide Frequency Range of 10^{-2}–10^{10} Hz

Kai-Lun Zhang, Na Li, Xu Li, Jun Huang, Haosen Chen, Shuqiang Jiao* and Wei-Li Song**

Supporting Information

Understanding enhanced ionic conductivity in composite solid-state electrolyte in a wide frequency range of 10^{-2} to 10^{10}Hz

Kai-Lun Zhang, Na Li, Xu Li, Jun Huang, Haosen Chen, Shuqiang Jiao*, Wei-Li Song**

K. L. Zhang, N. Li, Xu Li, Prof. H. Chen, Prof. W. L. Song, Prof. S. Jiao

Institute of Advanced Structure Technology, Beijing Institute of Technology, Beijing 100081, P. R. China.

E-mail: chenhs@bit.edu.cn; weilis@bit.edu.cn

Prof. S. Jiao

State Key Laboratory of Advanced Metallurgy, University of Science and Technology Beijing, Beijing 100083, P. R. China.

E-mail: sjiao@ustb.edu.cn;

Prof. J. Huang

Institute of Theoretical Chemistry, Ulm University, 89069, Ulm, Germany

Figure S1. Chronoamperometry (CA) spectrums of (a) PEO-TiO_2, (b) PEO-F(1)-TiO_2, (c) PEO-F(2)-TiO_2, (d) PEO-F(3)-TiO_2 and (e) PEO-F(4)-TiO_2 composite SSEs at 30 °C.

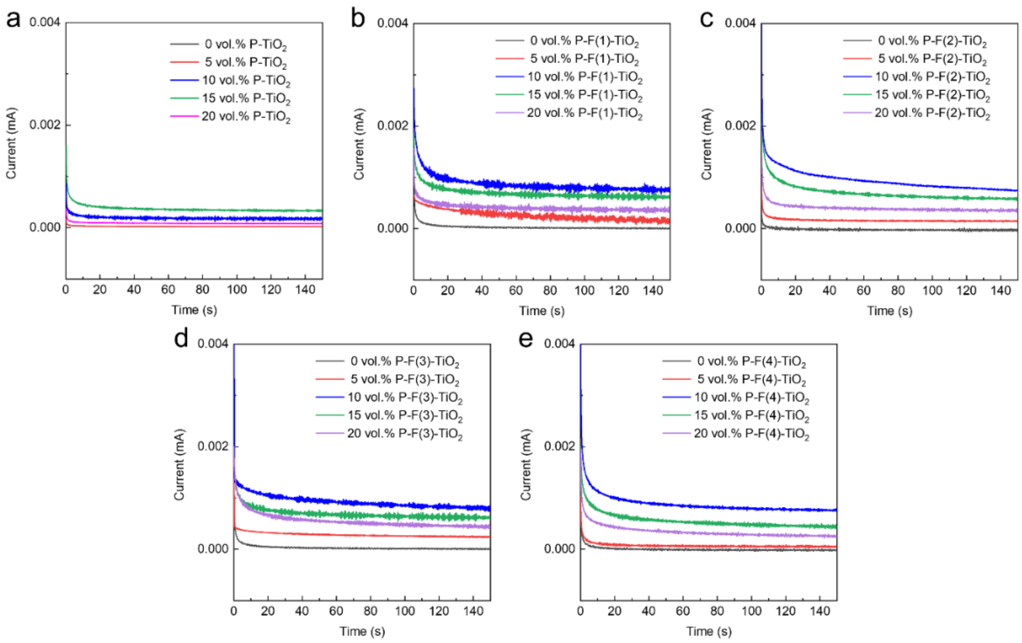

Figure S2. Electrochemical impedance spectrums (EIS) of (a) PEO-TiO_2, (b~f) PEO-F(1)-TiO_2, (g~k) PEO-F(2)-TiO_2, (l~p) PEO-F(3)-TiO_2 and (q~u) PEO-F(4)-TiO_2 composite SSEs at 30 ℃.

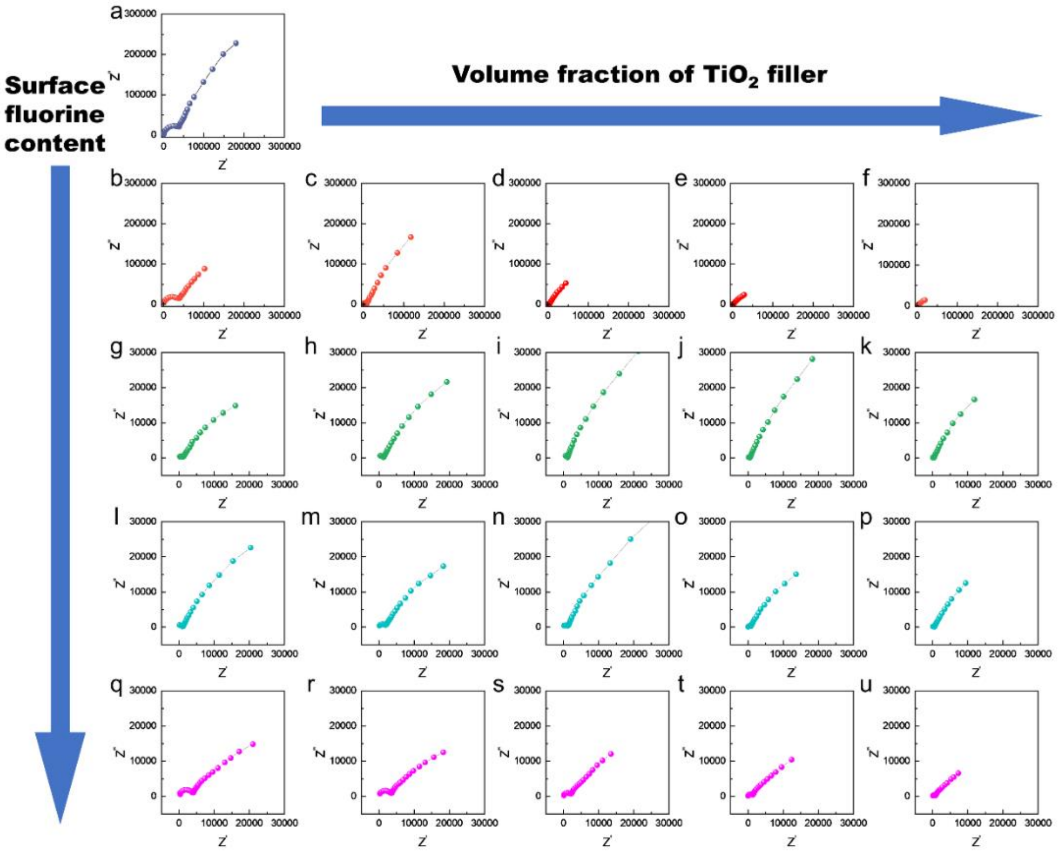

Figure S3. (a) Equivalent circuit for the analysis of EIS results (upper), where R_{sys} represents resistant of electrode and copper wire, C_{eff} is the effective capacitor of the structure capacitor of electrode C_g and capacitor of interface layer C_{int}, R_i is the resistance of ion conduction, W represents the Warburg impedance, the equivalent circuit could be simplified due to a ignoreable small resistance of copper wire and large resistance of electrolyte (bottom); ionic conductivity of (b) PEO-TiO_2, (c) PEO-F(1)-TiO_2, (d) PEO-F(2)-TiO_2, (e) PEO-F(3)-TiO_2 and (f) PEO-F(4)-TiO_2 composite SSEs.

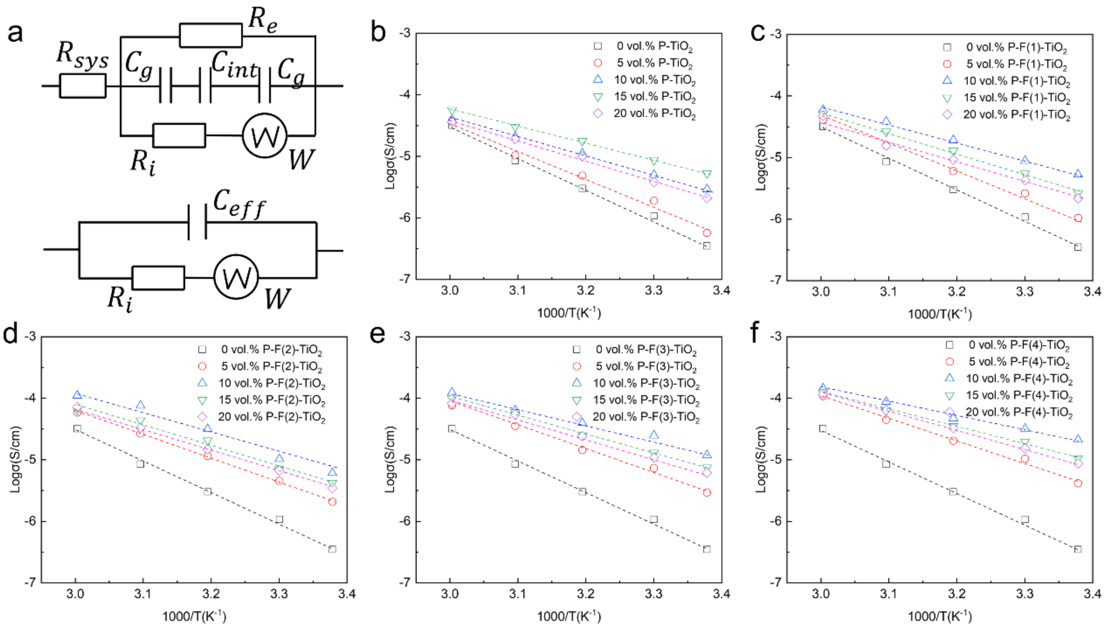

Figure S4. Dielectric spectrums (DS) of (a) PEO-TiO_2, (b) PEO-F(1)-TiO_2, (c) PEO-F(2)-TiO_2, (d) PEO-F(3)-TiO_2 and (e) PEO-F(4)-TiO_2 composite SSEs at 30 °C.

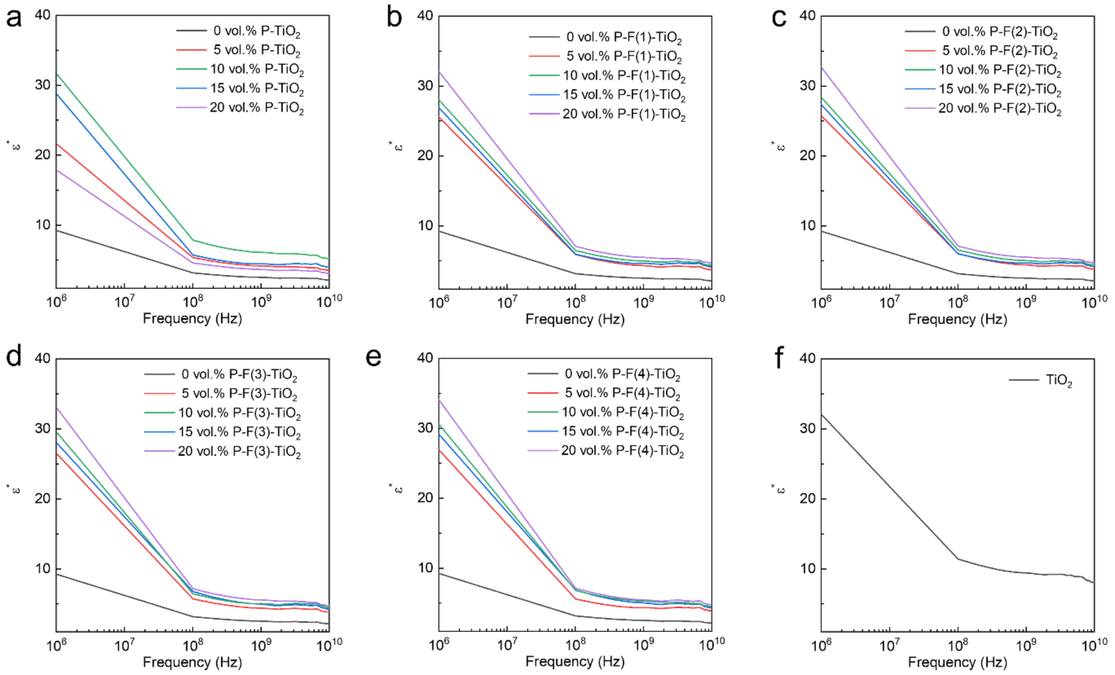

Table S1. Used parameters in Eq. 6.

Smaple	σ_0	w	α	β	$\sigma_I : \sigma_B$
P-TiO_2	0.000001	0.000025	3	2	1:7
P-F(1)-TiO_2	0.000001	0.000025	3	3	1:10
P-F(2)-TiO_2	0.000001	0.000025	3	2.5	1:12
P-F(3)-TiO_2	0.000001	0.00006	3	2.5	1:13
P-F(4)-TiO_2	0.000001	0.000075	3	2.5	1:14

Table S2. Unsteady current of Chronoamperometry.

Smaple	unsteady current of CA (mA)	Signal to Noise Ratio (%)
10 vol. % P-TiO_2	2.13×10^{-7}	1.06
10 vol. % P-F(1)-TiO_2	1.56×10^{-5}	2.49
10 vol. % P-F(2)-TiO_2	4.38×10^{-6}	0.75
10 vol. % P-F(3)-TiO_2	7.01×10^{-6}	1.22
10 vol. % P-F(4)-TiO_2	6.51×10^{-7}	0.35

Calculation of carrier concentrion *c*:

The diffussion coefficient *D* can be caculated from Warburg impedance:[1]

$$D = \frac{(RT)^2}{z^2F^4c^2S^2B^2}, \quad (1)$$

where *S* is the area of ion-blocking electrodes, *B* Warburg coefficient derived from electrochemical impedance spectrum(EIS), *c* carrier concentration, *z* the valence electron number of ion. *F*, *R*, and *T* are Faraday constant, gas constant and temperature, respectively. Quasi-quilibrium approximation is suitable for the measuring system of chronoamperometry (CA) and EIS due to small amplitude of signal. The relationship between D and conductivity can be descript by Nernst-Einstern equation:[2]

$$\sigma_{CA} = D\frac{F^2c}{RT}, \quad (2)$$

where is the σ_{CA} is the diffusion-dominated ionic conductivity calculated from CA.[43] z = 1 for conducting lithium-ions. After combining Eqs. (1) and (2), carrier concentration can be calculated according to:

$$c = \frac{RT}{2F^2\sigma_{CA}S^2B^2}, \quad (3)$$

Reference:

[1] J. Huang, *Electrochim. Acta* **2018**, *281*, 170.

[2] R.A. Mckee, *Solid State Ionics* **1981**, *5*, 133.
